# Quality of life and participation in daily life of adults with Pompe disease receiving enzyme replacement therapy: 10 years of international follow-up

**DOI:** 10.1007/s10545-015-9889-6

**Published:** 2015-11-03

**Authors:** Deniz Güngör, Michelle E. Kruijshaar, Iris Plug, Dimitris Rizopoulos, Tim A. Kanters, Stephan C. A. Wens, Arnold J. J. Reuser, Pieter A. van Doorn, Ans T. van der Ploeg

**Affiliations:** Department of Neurology, Center for Lysosomal and Metabolic Diseases, Erasmus MC University Medical Center, PO BOX 2060, 3000 CB Rotterdam, The Netherlands; Department of Pediatrics, Center for Lysosomal and Metabolic Diseases, Erasmus MC University Medical Center, Rotterdam, The Netherlands; Department of Biostatistics, Erasmus MC University Medical Center, Rotterdam, The Netherlands; Department of Health Policy and Management, Institute for Medical Technology Assessment, Erasmus University Rotterdam, Rotterdam, The Netherlands; Department of Neurology, Erasmus MC University Medical Center, Rotterdam, The Netherlands; Department of Clinical Genetics, Erasmus MC University Medical Center, Rotterdam, The Netherlands

## Abstract

**Background:**

Pompe disease is an inheritable metabolic disorder for which enzyme replacement therapy (ERT) has been available since 2006. Effects of ERT have been shown on distance walked, pulmonary function and survival. We investigated whether it also improves quality of life and participation in daily life in adult patients with the disease.

**Methods:**

In an international survey, we assessed quality of life (Short Form 36, SF-36) and participation (Rotterdam Handicap Scale, RHS) annually between 2002 and 2012. Repeated measurements mixed effects models were used to describe the data over time.

**Results:**

Responses were available for 174 adult patients. In the periods before and after start of ERT, the median follow-up times were 4 years each (range 0.5-8). The SF-36 Physical Component Summary measure (PCS) deteriorated before ERT (-0.73 score points per year (sp/y); CI 95 % -1.07 to -0.39), while it improved in the first 2 years of ERT (1.49 sp/y; CI 0.76 to 2.21), and remained stable thereafter. The Mental Component Summary measure (MCS) remained stable before and during ERT. After declining beforehand (-0.49 sp/year; CI -0.64 to-0.34), the RHS stabilized under ERT.

**Conclusion:**

In adult patients with Pompe disease, ERT positively affects quality of life and participation in daily life. Our results reinforce previous findings regarding the effect of ERT on muscle strength, pulmonary function and survival.

## Introduction

Pompe disease (OMIM 232300) is an inheritable metabolic disorder caused by a deficiency of the lysosomal enzyme acid α-glucosidase, which is needed to break down glycogen (Hirschhorn and Reuser 2001). Lysosomal glycogen consequently accumulates in cells throughout the body, particularly in muscle cells, resulting in a wide spectrum of symptoms and complications (Gungor and Reuser 2013). Typically, adult patients have skeletal and/or respiratory muscle weakness, as well as symptoms such as pain (Gungor et al 2013) and fatigue (Gungor et al 2013; Hagemans et al 2007b). Most eventually become wheelchair-bound or require respiratory support. They are at risk of succumbing to respiratory failure or insufficiency. The resulting impact of the disease on their quality of life (QoL) and their participation in daily life is substantial. Many patients are limited in their ability to work or study (Hagemans et al 2007a), and their physical health status has been shown to be less than that of people in the general population (Hagemans et al 2004; van der Ploeg et al 2010; Vielhaber et al 2011). On the other hand, their mental-health status was found not be reduced (Hagemans et al 2004; Kanters et al 2011; Wokke et al 2008).

Currently, enzyme replacement therapy with alglucosidase alfa is the only approved treatment for Pompe disease. By improving and stabilizing skeletal muscle strength and function, and respiratory function, this lifelong treatment has improved the prospects of adult patients with the disease (Gungor et al 2013; Toscano and Schoser 2013; van der Ploeg et al 2010). Most importantly, ERT improves survival (Gungor et al 2013). However, it is less clear whether it also improves patients’ quality of life and participation, especially after longer treatment. The only placebo-controlled clinical trial to have assessed the efficacy of ERT in 90 adult patients showed that physical health status (measured by the SF-36) after 18 months of ERT did not differ between the placebo group and the treatment group (van der Ploeg et al 2010). And while the only observational study to include quality of life (measured using the SF-36) in its assessment of the effects of ERT indicated that QoL remained stable during treatment (Regnery et al 2012; Strothotte et al 2010), several individual case reports and case series reported improvements (Merk et al 2009; Orlikowski et al 2011; Vielhaber et al 2011; Angelini et al 2009).

To the best of our knowledge, no study in large groups of patients has determined whether ERT affects longer-term Qol. Neither has the effect of ERT on participation been studied. To fill these gaps in knowledge, we studied the QoL and participation in daily life of adults with Pompe diseases before and after long-term treatment with ERT.

## Methods

### Data collection

This study was performed as part of an ongoing prospective observational cohort study in patients with Pompe disease that started in May 2002. Information was collected through annual surveys sent out by mail and, since May 2009, also collected through a secure web-interphase. Questionnaires covered data on medical history, patient demographics, quality of life and participation in daily life (Hagemans et al 2005a, b). Patients from the Netherlands, the United States, the United Kingdom, Germany and Australia were recruited through the national patient organizations, as were a smaller number of patients from other countries. In addition, Dutch patients were also recruited through the Erasmus MC University Center for Lysosomal and Metabolic Diseases, the national referral center for Pompe disease. Data collection for the current study was locked in February 2012; it included only adult patients aged 18 years and older who had started treatment at some point during follow-up. To be included in the analyses, patients had to have been receiving ERT for at least 6 months, and also to have had a minimum of 6 months follow-up before the start of ERT. The study was approved by the ethics committee of the Erasmus MC University Medical Center and written informed consent was obtained from all participants

### Measurement scales

#### Quality of life

Quality of life was measured using the Medical Outcome Study 36-item Short Form Health Survey (SF-36), a generic instrument that has been widely used, has been translated into many languages, and has been shown to have good reliability and validity (McHorney et al 1993; Wagner et al 1998; Ware and Sherbourne 1992). It comprises eight domains: physical functioning, role physical, bodily pain, general health, vitality, social functioning, role emotional and mental health. Items are summed per domain and transformed into scores between 0-100, with higher values representing better function. Two summary scores can also be derived from the SF-36: the physical component summary measure (PCS) and the mental component summary measure (MCS). Norm-based scores were calculated using the US 1998 norm-based scoring for all patients; this is common practice, as the differences with country-based norm-scores have been shown to be very small for data collected in Western European countries (Ware et al 1998). Norm-based scoring assures that results for both versions of the SF-36 can be directly compared. In our study, we used SF36 version 1.0 between 2002 and 2009, and version 2 in the years thereafter.

#### Participation in daily life activities

To assess the level of Participation, which is defined as a person’s involvement in life situations (previously called ‘Handicap’) (WHO 1980, 2001), we used the Rotterdam Handicap Scale (RHS). The RHS is a brief measurement scale consisting of nine items covering mobility, domestic tasks and leisure activities (each assessed indoors and outdoors), as well as kitchen tasks, driving a car/going by bus/ride a bicycle and work/study. The scores per item range from 1 (‘unable to fulfil the task or activity’) to 4 (‘complete fulfilment of the task or activity’). Patients can also answer that an item does not apply to them, in which case a score of 0 is given. The total score is calculated as the sum of the item scores divided by the number of applicable items and multiplied by 9 (Hagemans et al 2007a; Merkies et al 2002). The RHS score thus ranges from 9 to 36. In the present analysis, no score was calculated if 4 or more items were non-applicable or missing. As a result, 2 % of measurements (32/1358) were excluded from analyses.

### Statistical analyses

The longitudinally assessed outcome scores (PCS and MCS of the SF-36; RHS) were analysed using mixed effects models, which allowed for irregular measurement times and different treatment/follow-up durations, including the fact that not all patients had measurements that dated from exactly the start of ERT. In the fixed-effects part, piece-wise linear regression was used to assess the mean annual change in the outcome scores in different time periods; the scores were expressed in absolute score points (sp/y). Whenever it was possible to assume linearity in the changes in outcome scores within the periods before and after the start of ERT, we used the ‘broken-stick method’, in which the breakpoint was chosen at the time that ERT was started. Linearity was statistically assessed on the basis of inclusion of a quadratic term for time (p < 0.05) and also by inspecting the Loess nonparametric smoother. If the changes in outcome scores before or after ERT were not linear, this non-linear time period was divided into two smaller linear segments, in which further breakpoints were placed at 2.0 years of ERT, based on our clinical knowledge and the Loess nonparametric smoother.

Subgroup analyses were performed to assess whether a treatment effect, if any, differed between more and less severely affected patients. Disease severity was defined as wheelchair use (yes/no) and use of respiratory support (yes/no). Using the likelihood ratio test we first assessed whether adding a subgroup significantly improved the mixed effects model (p < 0.05). If so, the variable, and its interactions with time were included in the model and interpreted.

Data analyses used SPSS for Windows (version 17, SPSS Inc., Chicago, IL) and SAS (version 9.2, SAS Institute Inc., Cary, NC). A p-value of ≤ 0.05 was considered statistically significant.

## Results

### Patients

**Fig. 1:**
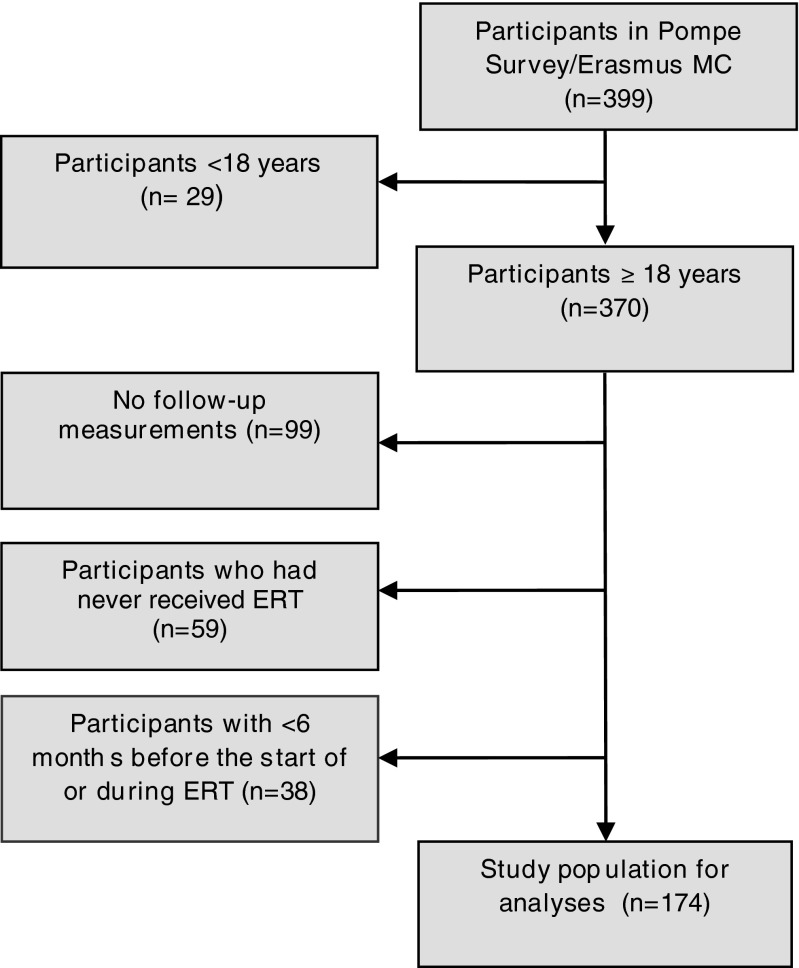
Flowchart of the study population for the analyses

One hundred and seventy-four adult Pompe patients were available for the current analysis. Figure 1 shows the inclusion process. There were three reasons for excluding adult patients: not receiving ERT (n = 59), no follow-up measurements available (n = 99) and less than six months follow-up either before or after the start of ERT (n = 38). At baseline, excluded patients were comparable to those included in the analysis in terms of gender and disease severity, but not with regard to age. Excluded patients were slightly older (median 51 years, (range 20-81 years) vs 46 years (range 19-72 years; p = 0.001)).

Table 1 shows the characteristics of the study population. Fifty-three percent of the participants were women. Patients’ median age at start of ERT was 50 years (range 24-76); 52 % used a wheelchair, and 48 % required respiratory support. The median total follow-up time was 7 years (range 1-10), with a median follow-up period of 4 years (range 0.5-8) both before and during ERT.

**Table 1:** Characteristics of the study population

Demographics	Study population *N* = 174
Age at start of ERT, median (range)	50 (24–76)
Age at diagnosis, median (range)	37 (1–66)
Female gender, no. (%)	93 (53)
Country of residence, no. (%)	
Netherlands	64 (37)
Germany	36 (21)
US	39 (22)
UK	16 (9)
Other	19 (11)
Clinical characteristics	
Disease duration, median (range)	12 (1–33)
Use of wheelchair at start of ERT, no. (%)	90 (52)
Respiratory support at start of ERT, no. (%)*	84 (48)
Follow-up during study	
Total follow-up time, median (range)	7 (1–10)
Pre-treatment period, median (range)	4 (0.5–8)
Treatment period, median (range)	4 (0.5–8)

Age, disease duration and follow-up time are expressed in years as median (range). Categorical variables are expressed as number (no.) and percentage (%);

*‘Respiratory support’ includes partial and fulltime, invasive and non-invasive respiratory support

At start of ERT, patients had a median PCS score of 31, well below the general population norm of 50, while their median MCS score of 54 was within the normal range. Patients’ had a median RHS score of 25 at the start of ERT, which was below the maximum score of 36 that would be scored by an average healthy person.

### Effect of ERT on quality of life

During ERT, PCS and MCS scores did not follow a linear pattern over time. We therefore distinguished two time intervals during ERT: 1. from start of ERT to two years of ERT, 2. and more than two years of ERT (see Table 2).

**Table 2:** SF-36 quality of life scores before and during ERT

Clinical outcome measure	Follow-up time intervals (in years)		
SF-36 Component summary scores	Pre-ERT Mean sp/y (95 % CI)	0 to 2 years during ERT Mean sp/y (95 % CI)	>2 years during ERT Mean sp/y (95 % CI)
Physical component summary	-0.73 (-1.07; -0.39)**	1.49 (0.76; 2.21)**	-0.15 (-0.43; 0.13)
Mental component summary	0.16 (-0.25; 0.57)	1.03 (-0.07; 2.13)	0.02 (-0.41; 0.46)
SF-36 domain scores			
Physical functioning	-1.80 (-2.41; -1.19)**	1.81 (0.38; 3.23)*	0.68 (0.12; 1.25)*
Role physical	-1.20 (-2.92; 0.52)	9.18 (5.83; 12.53)**	2.46 (1.15; 3.77)**
Bodily pain	-2.10 (-3.01; -1.19) **	0.76 (-1.39; 2.92)	-1.96 (-2.95; -0.98)**
General health	0.19 (-0.49; 0.87)	5.22 (3.58; 6.86)**	0.81 (0.13; 1.49)*
Vitality	-0.44 (-1.11; 0.24)	4.10 (2.44; 5.76)**	1.43 (0.77; 2.09)**
Social functioning	-0.74 (-1.66; 0.18)	2.11 (-0.16; 4.39)	0.41 (-0.54; 1.35)
Role emotional	-0.15 (-1.64; 1.34)	3.05 (-1.06; 7.16)	0.19 (-1.15; 1.53)
Mental health	0.13 (-0.60; 0.87)	2.09 (0.57; 3.61)**	0.29 (-0.37; 0.95)
Rotterdam handicap scale score	Pre-ERT Mean sp/y (95 % CI)	During ERT Mean sp/y (95 % CI)	
	-0.49 (-0.64;-0.34)**	-0.02 (-0.17; 0.13)	

Data shown are mean changes in score points per year (sp/y) as calculated by univariate analysis using mixed model ANOVA

*CI* confidence interval

*Change is significant at the 0.05 level, **Change is significant at the 0.01 level

In the period before starting ERT, the PCS decreased significantly by 0.73 score points per year (sp/y; CI 95 % -1.07 to -0.39). This was followed in the first two years after treatment by a significant increase of 1.49 sp/y (CI 95 % 0.76 to 2.21), and by stabilization thereafter (-0.15 sp/y; CI 95 % -0.43 to 0.13). The MCS was more or less stable during the entire follow-up period.

Table 2 also shows the separate changes in the eight health domains of the SF36. Throughout treatment follow-up, the physical functioning, role physical, general health and vitality domains increased, with particularly large increases during the first two years of ERT in role physical (9 sp/y), general health (5 sp/y) and vitality (4 sp/y). Mental-health domain scores increased until 2 years of ERT (2 sp/y), while bodily pain, social functioning and role emotional did not increase during treatment. In fact, bodily pain seemed to deteriorate in the later years of ERT, as it had before starting ERT.

**Fig. 2:**
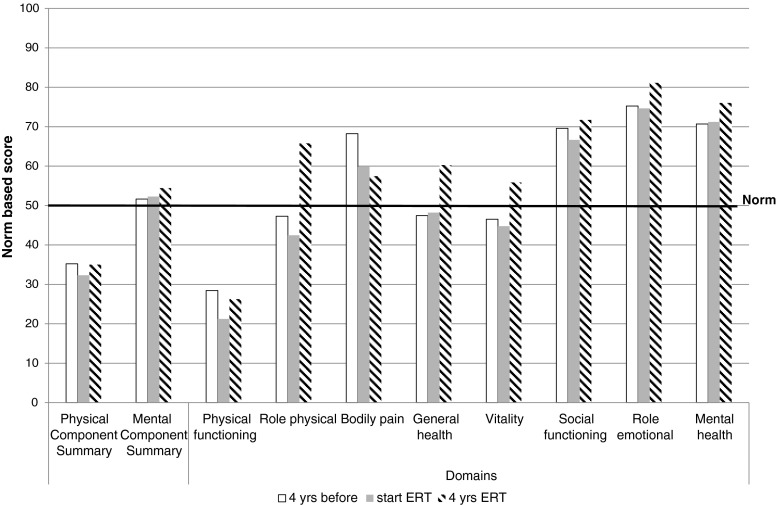
Expected SF-36 quality of life scores before, at start and during ERT. The figure displays the expected mean scores 4 years before starting ERT, at start of ERT and after 4 years of ERT as calculated using the results from the statistical model presented in Table 2

Using the results from the statistical analysis presented above we calculated the expected SF-36 scores at three time-points: 4 years before ERT, at start of ERT and after 4 years of ERT (Fig. 2). Before starting treatment, the PCS and the domains physical functioning, role physical and vitality were below the general population norm. While all four improved with treatment, the PCS and the physical functioning domain scores remained below the population norm after 4 years of ERT.

Subgroup analyses showed that less severely affected patients (i.e. those not using a wheelchair or not using a ventilator) overall had better PCS scores than more severely affected patients (p < 0.01). MCS scores did not differ between these two groups. Subgroup analyses did not show any differences in the effect of ERT on the PCS or MCS scores between more and less severely affected patients.

### Effect of ERT on participation in daily life

Because with regard to the RHS the changes in the periods before and after the start of ERT could be assumed to be linear, the breakpoint was set only at the start of ERT. Before starting ERT, RHS scores decreased significantly by a mean of 0.49 sp/y (CI 95 % -0.63 to -0.34). During ERT, however, they stabilized (-0.02 sp/y; CI 95 % -0.17 to 0.13; Table 2). Subgroup analyses showed that less severely affected patients had better RHS scores overall, but it did not show any differences in the effect of ERT on participation between more and less severely affected patients.

## Discussion

This study in a large international cohort of patients with Pompe disease showed enzyme replacement therapy to have a positive effect on patients’ physical health status (PCS) and their participation in daily life. The use of ERT halted the progressive decline in physical health status and participation observed pre-treatment; during the first two years of ERT patients’ physical health status even improved. Patients’ mental-health status (MCS) remained the same throughout the entire follow-up including the pre-treatment period.

The significant improvement in the PCS during the first two years of treatment was most noticeable in the role physical domain, which increased by 9 score-points per year, reflecting a large increase in patients’ ability to perform their work and other common activities. Two physical health domains were below the population norm in our Pompe cohort: physical functioning and role physical (see also Hagemans et al 2004). These domains improved over the entire treatment follow-up, as did general health, explaining the increase in PCS scores after the start of ERT.

The improvements in the physical functioning and role physical domains may have been due to the positive effect of ERT on muscle function and strength seen in other studies (Angelini et al 2012; Bembi et al 2010; de Vries et al 2012; Orlikowski et al 2011; Regnery et al 2012; van der Ploeg et al 2012; van der Ploeg et al 2010). In addition to the three domains that improved, bodily pain is important to determining the PCS level. In the first two years after the start of ERT, bodily pain stabilized, but with longer treatment it declined, indicating more pain. This may signify that ERT is only partially curative. Only one other study (in two patients) has provided information on the effect of ERT on pain; this showed that pain decreased in the year after the start of ERT (Vielhaber et al 2011). Recently, pain was also shown to be a relatively common symptom in treated and untreated patients alike (Gungor et al 2013).

Ours is the first study in adult Pompe patients to examine the effect of ERT on their participation in daily life. Patients’ reduced physical health status at start of ERT was mirrored by their lower level of participation in daily life. The impact of Pompe disease on participation and the decline in participation over time before ERT has previously been described in a group of 40 Dutch patients (Hagemans et al 2006), and was confirmed by the current study. Compared to the decline observed prior to ERT, the stabilization that took place during treatment should be seen as an important effect of therapy: for patients with Pompe disease, it is very valuable to maintain rather than lose a certain level of social activities.

Our observation that the MCS remained stable even without treatment is consistent with the outcomes of previous studies showing that adult Pompe patients have the same mental-health status as healthy individuals (Hagemans et al 2004; Kanters et al 2011). Over time, patients with a chronic disease such as Pompe disease may adapt to their situation by changing their standards, values and conceptualizations; this may explain their stable mental-health scores during follow-up. During ERT, there was an improvement in only one of the four domains that chiefly determine the MCS: vitality, which assesses the level of energy. This corroborates previous findings in this cohort in which ERT reduced the level of fatigue measured by the Fatigue Severity Scale (Gungor et al 2013).

As shown in earlier analyses of this survey, more severely affected patients had worse physical health and handicap scores than those less severely affected (Hagemans et al 2004 and Hagemans et al 2007a). Nevertheless, no difference was observed between these groups in the effect of ERT on these outcome measures. While this could suggest that ERT is equally beneficial in the experience of the patient, the results of these subgroup analyses should be interpreted with caution as we could not adjust for other factors that may differ between these patients.

This is the first time that significant positive effects of ERT on physical health status (PCS) have been demonstrated in such a large study with long follow-up, although they have been suggested by some other studies (Orlikowski et al 2011; Toscano and Schoser 2013). There are two possible reasons why such positive effects were not demonstrated by the pivotal 18-month placebo-controlled trial by van der Ploeg et al on the effects of ERT (van der Ploeg et al 2010). Firstly, while the original trial had a smaller study population, with 60 patients in the treatment arm and 30 in the placebo arm, our own study followed 174 patients both before and after the start of therapy. Secondly, while follow-up in the original trial was 78 weeks, median follow-up in our study was 4 years before start of ERT and 4 years afterwards. The fact that sufficient follow-up time and a large number of patients are required was also demonstrated by our earlier study of 38 untreated Dutch patients, a limited study with only one year of follow-up in which we found no significant deterioration in physical health status (Hagemans et al 2004). The same population was included in the current study: now, with a long pre-treatment period and a larger study group, the deterioration in PCS in untreated patients became significant.

Randomized controlled trials are generally seen as the gold standard for assessing the effects of treatment. However, while they are an important means of showing the short-term effect of drugs, their limited duration does not allow for the assessment of long-term treatment effects. Our own longitudinal survey supplements the evidence obtained from such trials by enabling the long-term treatment effects to be evaluated in a broad patient population that also includes severely affected patients who would normally be excluded from a trial. It is also the case that international surveys and registries are the only way to obtain sufficient patient numbers for rare disorders such as Pompe disease. Our study thus has the advantage of incorporating a large cohort of Pompe patients of different ages and severities across different countries over a long follow-up period (up to 10 years).

The recruitment of patients through patient organizations might be seen as a limitation of this study, as it could result in the exclusion of patients at the more or less severe end of the spectrum. However, the demographic and clinical characteristics of our study population show that patients were included across the entire spectrum of adult Pompe disease. Although the SF-36 is a generic instrument that may not necessarily cover all the domains that are relevant in Pompe disease, we chose to use it because it is readily available and is also widely used in many different disorders. While more comprehensive information could be derived by using a disease-specific scale such as the R-Pact (van der Beek et al 2013), the R-Pact was not available when the survey was started.

Because patients’ perceptions of their health-related quality of life and participation in daily life can provide important information about the impact of a disease on patients’ lives, they are increasingly used in clinical research and practice. In 2006, the Food and Drug Administration (FDA) and The European Agency for the Evaluation of Medicinal Products (EMEA) both acknowledged the importance of such outcomes (US. FDA 2009; European Medicine Agency 2004). In the study of Pompe disease, the chronic progressive character of the disease means that patients will probably need lifelong therapy. While patients’ lives might be negatively affected by the possible burden of bi-weekly infusions, our results show that this burden is outweighed by the positive effects of ERT.

In conclusion ERT positively affects quality of life and participation in daily life of adult Pompe patients. Before treatment, physical health status and participation declined progressively. After the start of ERT, both stabilized. The effect on physical health status seemed to be greatest in the first two years after ERT. The mental-health status of these patients did not seem to be affected. These results reinforce the evidence available on the efficacy of ERT, showing that as well as its beneficial effect on muscle strength, pulmonary function and survival, ERT also improves patients’ perceived participation and quality of life.

## Glossary

ERT: Enzyme replacement therapy
MCS: Mental Component Summary measure
PCS: Physical Component Summary measure
QOL: Quality of Life
RHS: Rotterdam Handicap Scale
SF-36: Medical Outcome Study 36-item Short Form Health Survey
